# Complete chloroplast genome sequence of *Mucuna prurien* and its phylogenetic position

**DOI:** 10.1080/23802359.2021.1915199

**Published:** 2021-07-27

**Authors:** Xiao-Long Yuan, Dao-Xi Zha, Lin Xue, Xue-Ying Wang, Gang Xu, Wen-Bao Li, Hai-Qing Xu

**Affiliations:** a Tobacco Research Institute of Chinese Academy of Agricultural Sciences, Qingdao, PR China; b Wannan Tobacco Group Co., Ltd, Xuancheng, PR China

**Keywords:** *Mucuna pruriens*, chloroplast genome, phylogenetic position

## Abstract

*Mucuna pruriens* is traditional medicinal plant originated in South Africa. We characterize the complete plastid genome of *M. pruriens*, which is a circular-mapping molecule 152,119 bp in length. The genome has a large single-copy region (LSC) of 78,258 bp and a small single-copy region (SSC) of 18,735 bp, respectively. Additionally, the overall GC content of the chloroplast genome was 35.37%. The genome contains 138 genes, including 96 protein-coding, 38 *tRNA*, and four *rRNA* genes. The gene content and structure are conserved compared to other species in the genus *Glycine*. The chloroplast genome and existing data were used to infer its phylogenetic position. The results showed that *M. pruriens* clustered together with *Glycine max* and *G. soja*. These findings provide potential genetic markers that can aid in understanding the genetic diversity of *M. pruriens*.

*Mucuna pruriens* (Papilionoideae), known as velvet bean, originated in South Africa. Currently, there is interest in its abundant nutrients and the function of its phytochemical constituents owing to its excellent medicinal value (Herrera-Chalé et al. 2016). Although *M. prurien* has high nutritive and medicinal value, no literature on the genome of *M. pruriens* has been published. The chloroplast is a vital organelle that participates during photosynthesis and the synthesis of secondary metabolites, which can serve as molecular makers to infer the phylogeny of species (Verbruggen et al. 2010). Herein, we characterize the complete chloroplast genome of *M. pruriens* and infer its phylogenetic position.

Fresh, young leaves of *M. pruriens* (specimen number: 20190728MG01) were collected from Xuancheng, Anhui Province of China (118′598′′, N 30′778′ E, 180 m). The specimen was stored at the herbarium of the Bank of Special Groups Germplasm Resources of Chinese Academy of Agricultural Sciences, Qingdao, China.

The experiment procedure is as reported in Gao and Gao (2017). Around 2.6 Gb of clean reads were assembled against the plastome of proximal species (*Glycine gracilis*, *Glycine max*, and *Glycine soja*) using SOAPdenovo (Luo et al. 2012). Geneious version 11.0.2 (Kearse et al. 2012), coupled with the NCBI ORF-finder (http://www.ncbi.nlm.nih.gov/gorf/gorf.html) were used to predict and annotate the mitochondrial genome of *M. pruriens*.

The complete *M. pruriens* (GenBank accession number MT919240) chloroplast genome is 152,119 bp in size and contains a large single-copy region (LSC) of 78,258 bp and a small single-copy region (SSC) of 18,735 bp, which were separated by a pair of inverted repeat (IR) regions of 27,563 bp. Additionally, the overall GC content of the chloroplast genome of *M. pruriens* was 35.37%, and the corresponding values of the LSC, SSC, and IR regions were 34.32%, 36.14%, and 51.16%, respectively. The genome contains 138 genes, including 96 protein-coding, 38 *tRNA*, and four *rRNA* genes.

In total, 13 chloroplast genomes from legumes were used to construct phylogenetic relationships using MrBayes version 3.2 (Huelsenbeck and Ronquist 2001). The results showed that *M. pruriens*, *Glycine max*, and *G. soja* clustered in a unique clade in the *Phoebe* genus (Figure 1). The complete chloroplast genome enables us to understand its genomic features and provides useful gene markers to elucidate the evolution of the *Mucuna* genus.

**Figure 1.**
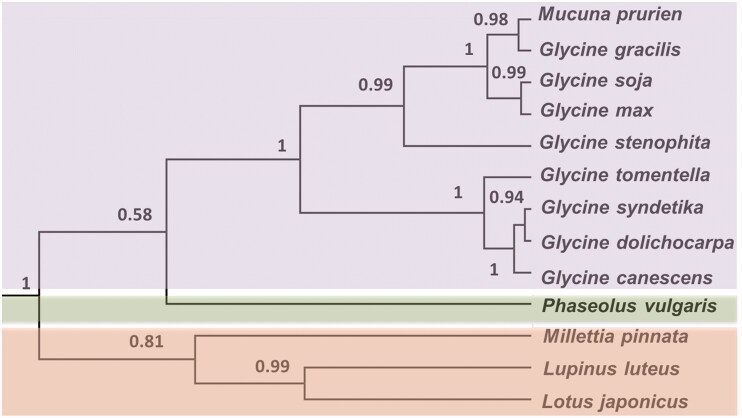
Phylogenetic tree of *M. pruriens* with 12 species was constructed by chloroplast plastome sequences. Four simultaneous chains were run for 10,000,000 generations.

## Data Availability

The data that support the findings of this study are openly available in GenBank of NCBI at https://www.ncbi.nlm.nih.gov, reference number MT919240. Meanwhile, the raw data of sequencing has been deposited to NCBI with project number: PRJNA665117.
